# Heparin-Induced Thrombocytopenia: A Case Report Four Years After the Initiation of Maintenance Hemodialysis

**DOI:** 10.7759/cureus.52094

**Published:** 2024-01-11

**Authors:** Junichiro Nishiyama, Keishi Higashi

**Affiliations:** 1 Nephrology, Seisyukai Clinic, Fukuoka, JPN; 2 General Internal Medicine, Kusumoto Medical Clinic, Fukuoka, JPN

**Keywords:** heparin induced thrombocytopenia, hemodialysis, maintenance hemodialysis, hit antibody, argatroban, plasma exchange, thrombocytopenia, heparin, low molecular weight heparin (lmwh), disseminated intravascular coagulation (dic)

## Abstract

Heparin-induced thrombocytopenia type II (HIT) is a rare and serious complication of heparin exposure and is always a potential risk in hemodialysis patients who routinely receive heparin. It is particularly likely to occur during the induction phase of dialysis. However, it is known to be less prevalent in long-term maintenance dialysis. In the present study, we experienced a maintenance dialysis patient who developed HIT four years after starting dialysis. After careful diagnosis with antibodies assay and clinical scores, the patient was treated with immediate heparin interruption, argatroban administration followed by nafamostat, and simple plasma exchange, which resulted in remission. Therefore, even in the maintenance phase of hemodialysis, it is important to consider HIT in the differential diagnosis of thrombocytopenia.

## Introduction

Heparin-induced thrombocytopenia type II (HIT) is a rare and serious complication in patients exposed to heparin, with a potentially fatal outcome if not treated appropriately [1]. Hemodialysis patients who are routinely given heparin are at high risk of developing this condition. HIT usually develops five to ten days after heparin administration [2], so the incidence is particularly high during the induction phase of hemodialysis (3.9%) [3]. However, once patients enter the maintenance phase of dialysis, the incidence of HIT decreases drastically. In an analysis of 10,564 hemodialysis cases in the United Kingdom, the incidence was 0.26%, and even the longest interval after initiation of hemodialysis was 390 days [4]. We report here a case of HIT that occurred four years after induction of hemodialysis.

## Case presentation

**Figure 1:**
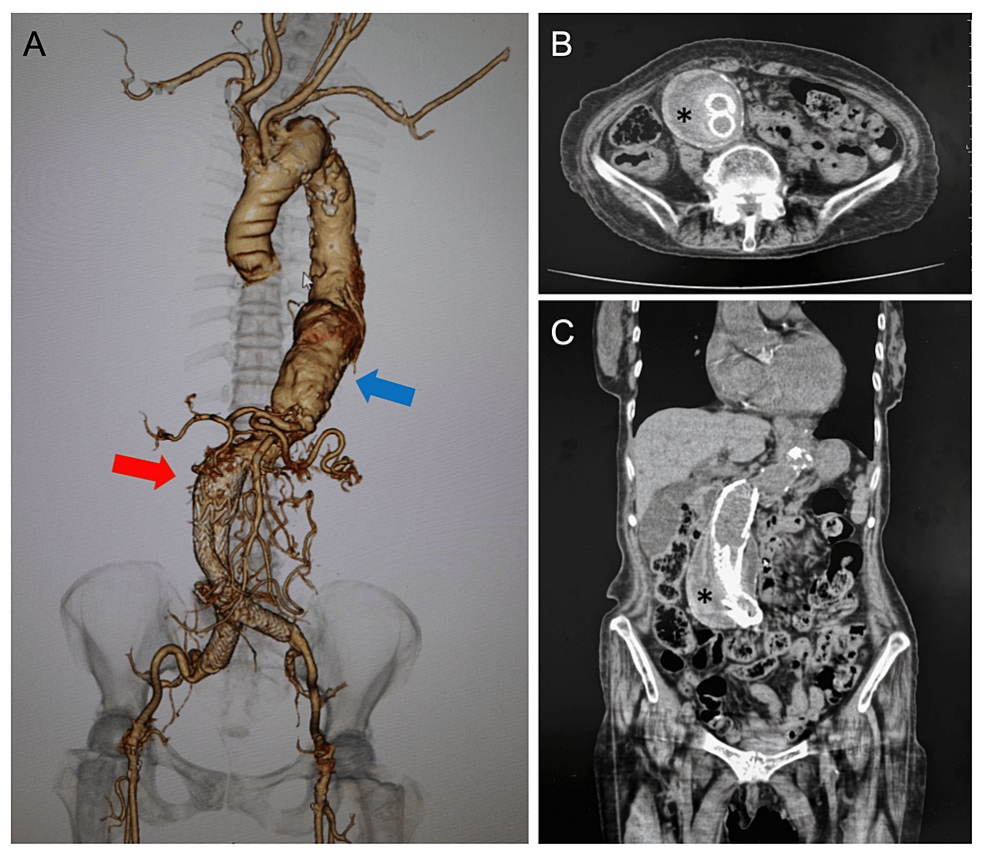
Computed tomography A: Contrast-enhanced computed tomography (CT) reconstruction image. Untreated thoracic aortic aneurysm, 54mm diameter (blue arrow), and abdominal aortic aneurysm after stent graft insertion (red arrow). B: CT cross-sectional image, C: CT coronal section image. Abdominal aortic aneurysm after stent graft insertion, 60 mm diameter aneurysmal sac (asterisk).

Four years ago, a female in her 80s developed end-stage renal disease and was started on hemodialysis, although her primary renal disease was unknown. She had been using dalteparin, a low molecular weight heparin, as an anticoagulant for hemodialysis since its introduction. From mid-September she began complaining of nausea several minutes after the start of each hemodialysis session, sometimes accompanied by vomiting. She also began to experience frequent drops in blood pressure during dialysis, which had rarely occurred before. At the same period, a mild worsening of anemia and marked thrombocytopenia (from 112,000/μL to 54,000μL) were observed. There was no bleeding tendency or thrombotic symptoms. She had various medical histories, including amputation of the right fifth toe due to cholesterol embolism five years ago and stent graft insertion for abdominal aortic aneurysm (AAA) four years ago followed by induction of hemodialysis. In the same year, she developed right abducens nerve palsy of unknown cause. Two years ago, she underwent root embolization of the inferior mesenteric artery caused by a type II endoleak in the stent graft. Six months later, she was treated with percutaneous endoaneurysmal embolization for re-enlargement of the same site (Figure 1).

In May, she developed a right pontine hemorrhage that was managed conservatively without sequelae. In July, she developed a spontaneous left pneumothorax, which was relieved after the insertion of a chest tube. Her height was 152.5 cm and her weight was 41 kg, vital signs were blood pressure 140/74 mmHg, pulse 97/min, and body temperature 36.9 degrees Celsius. Her consciousness was clear. There were no significant physical findings except for the right internal strabismus. Considering the patient's symptoms and possible causes of thrombocytopenia, such as gastrointestinal bleeding, coagulopathy, hemolytic anemia, infection, and autoimmune disease, various tests were performed, which revealed a high HIT antibody (latex agglutination method; HemosIL® HIT-A) level of 5.1 U/mL (normal range <1.0 U/mL) (Table 1).

**Table 1:** Laboratory parameters The patient was a maintenance hemodialysis patient with regular testing, thus, results showing at the time of submission of HIT antibodies assay. WBC: white blood cell, RBC: red blood cell, MCV: mean corpuscular volume, PT-INR: prothrombin-international normalized ratio, APTT: activated partial thromboplastin time, AST: aspartate transaminase, ALT: alanine transaminase, LDH: lactate dehydrogenase, BUN: blood urea nitrogen, TIBC: total iron binding capacity, IgG: immunoglobulin G, IgA: immunoglobulin A, IgM: immunoglobulin M, CH50: 50% hemolytic complement activity, C3: complement component 3, C4: complement component 4

Test	Ref range and units	Values
WBC	3300-9000 /μL	3600
Neutrophil %	40.0-75.0%	45.6
Lymphocytes %	18.0-49.0%	35.9
Monocytes %	2.0-10.0%	2.6
Eosinophil %	0.0-8.0%	15.0
Basophil %	0.0-2.0%	0.9
RBC	380-500×10^4 ^/μL	275
Hemoglobin	11.5-15.0 g/dL	8.5
MCV	87-102 fl	95
Platelets	14.0-35.0×10^4 ^/μL	5.4
Reticulocytes %	0.4-1.9%	1.0
PT-INR	0.85-1.15	1.09
APTT	27.0-38.0 sec	34.7
D-dimer	0.00-0.72 μg/mL	15.50
Haptoglobin 1-1	43-180mg/dL	65
Total protein	6.7-8.3 g/dL	6.5
Albumin	4.0-5.0 g/dL	3.5
AST	13-33 U/L	18
ALT	6-30 U/L	7
LDH	119-229 U/L	245
BUN	8.0-22.0 mg/dL	31.1
Creatinine	0.40-0.70 mg/dL	2.61
Serum iron	40-180 μg/dL	48
TIBC	270-440 μg/dL	208
Ferritin	4.0-87.0 ng/mL	215
C-reacted protein	0.00-0.30 mg/dL	0.50
IgG	870-1700 mg/dL	1316
IgA	110-410 mg/dL	466
IgM	46-260 mg/dL	202
CH50	30-46 U/mL	36.9
C3	65-135 mg/dL	81
C4	13-35 mg/dL	20
Antinuclear antibody	<40 folds	80
HIT antibodies	<1.0 U/mL	5.1

Upper gastrointestinal endoscopy revealed only chronic atrophic gastritis. No other abnormalities were observed that could have caused the aforementioned thrombocytopenia. Contrast-enhanced computed tomography showed no pulmonary embolism. Vascular Doppler sonography for deep vein thrombosis was negative in the bilateral lower extremities.

**Figure 2:**
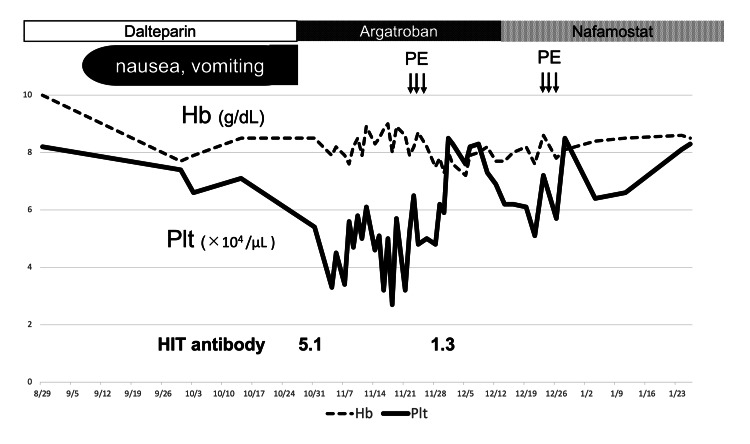
Clinical course In September, nausea and vomiting appeared with each hemodialysis session, and the patient’s anemia and thrombocytopenia worsened. After diagnosis of HIT, the anticoagulant was changed to argatroban. Her symptoms rapidly resolved, but thrombocytopenia exacerbated. Therefore, plasma exchange was performed twice. Eventually, thrombocytopenia improved. HIT: heparin-induced thrombocytopenia, PE: plasma exchange, Hb: hemoglobin, Plt: platelets

In addition, the clinical diagnostic score of HIT, 4T's (thrombocytopenia, timing of platelet count fall, thrombosis, other causes for thrombocytopenia) scores of 2, 1, 0, and 2 points, respectively, for a total of 5 points, that was in the POSSIBLE category, and the final diagnosis of HIT was determined. After diagnosis, the patient was considered for inpatient treatment, but there was no nearby hospital that could admit her because of the SARS-CoV-2 outbreak. Therefore, it was decided to perform a series of treatments on an outpatient basis. Immediately, the anticoagulant on dialysis was changed from dalteparin, a low molecular weight heparin, to argatroban. Argatroban was administered 10 mg intra-circuit at the start of dialysis and 16 mg/h during dialysis to maintain activated partial thromboplastin time (APTT) at approximately twice the normal level. Nausea during dialysis disappeared the day dalteparin was stopped and argatroban started. However, the thrombocytopenia gradually worsened. We tried to extend the duration of argatroban as an outpatient treatment option. In practice, the dialysis time was extended from four to six hours, and argatroban was administered for four hours on non-dialysis days. However, the platelet count continued to fall to 27,000/μL. Therefore, simple plasma exchange (PE) was implemented to remove residual HIT antibodies. Fresh frozen plasma was used as the replacement fluid, and the treatment volume was 2880 mL for three consecutive days. After treatment, HIT antibodies decreased to 1.3 U/mL and platelets increased. Due to a history of recent cerebral hemorrhage and previous AAA embolization, argatroban was discontinued on day 15 after PE considering the risk of argatroban per se with bleeding complications. Then the anticoagulant was changed from argatroban to nafamostat mesylate. It is a serine protease inhibitor with a very short half-life of five to eight minutes, making it a relatively safe anticoagulant for patients with bleeding tendencies. However, nafamostat is not approved by the FDA, but is mainly used in Japan and Korea. As the platelet counts gradually declined again, another PE was performed and a remission of the disease was achieved (Figure 2).

## Discussion

HIT is caused by the production of anti-platelet factor 4 (PF4)/heparin complex antibodies (HIT antibodies) following exposure to heparin. These antibodies activate platelets and monocytes, resulting in excessive thrombin production, thrombocytopenia, and arterial or venous thromboembolism. In addition, systemic reactions such as shivering, fever, hypertension, dyspnea, chest pain, nausea, and vomiting may also occur during heparin use, as in this case [5]. This is a serious complication with potentially high mortality without appropriate treatment [1]. In hemodialysis, heparin is widely used to prevent intra-circuit blood clotting during dialysis, so there is always a risk of HIT. It is particularly likely to occur during the induction phase of dialysis, and Yamamoto et al. reported an incidence rate of 3.9% [3].

However, in this case, the patient developed HIT more than four years after starting dialysis. Regarding HIT long-term after initiation of dialysis, there is a report by Matsuo et al. describing the onset of HIT 91 months after induction of dialysis [6] and a case report from Croatia describing the development of HIT 24 years after the start of dialysis [7]. There is also a large-scale British report [4]. According to this report, 28 (0.26%) of 10,654 hemodialysis patients developed HIT. The average time from the start of dialysis to the onset of HIT was 61 days, and even the longest case was 390 days after the induction of dialysis. Thus, HIT during maintenance dialysis is thought to be exceptional.

The diagnosis of this case was prompted by a positive HIT antibody test using the HemosIL® HIT-A latex agglutination assay. The sensitivity of this test is 100%, but the specificity is 84.3%, so a positive result does not confirm the diagnosis [8]. Similar immunoassays include ELISA, PaGIA, PIFA, CLIA, and lateral flow immunoassay [9]. Many of these assays, including the latex agglutination assay we used, have low specificity and only a fraction of patients who have a positive result develop HIT. This is partly because the assays react with IgM and IgA as well as IgG, and partly because only some of the anti-PF4/heparin complex IgG antibodies have pathological significance in activating platelets. In fact, about 10% of dialysis patients are antibody-positive, but only a small percentage develop HIT [10]. On the other hand, more accurate diagnostic functional tests, including 14C-serotonin release assay (SRA) and heparin-induced platelet activation assay (HIPA), are required for confirmation of the diagnosis. However, these functional tests are technically demanding, available in very limited facilities, and thus difficult to perform routinely. They were not available in this case. Therefore, in practice, the clinical score and antibody test results must be combined to confirm the diagnosis [2].

As the clinical score, 4T's score and HIT Expert Probability (HEP) score have been proposed. In this case, the diagnosis of HIT was determined based on the positive HIT antibodies and the clinical diagnosis of 4T's score of 5 points, meaning HIT POSSIBLE.

The patient had an elevated D-dimer level, which was not very compatible with HIT, but had remained high for at least four years since she was started on hemodialysis. It was speculated that the D-dimer elevation was attributable to an enhanced fibrinolytic system following continuous thrombus formation in the lumen of the aortic aneurysm. Although thrombosis is reported to occur in approximately 50% of HIT patients, there were no obvious thromboses in this case. HIT may present as simple thrombocytopenia without thrombosis, also known as isolated HIT, a condition that carries a significant risk of arterial and venous thrombosis [11].

When HIT is diagnosed, the first step is to discontinue heparin. Next, anticoagulation other than heparin is initiated to address the risk of thrombosis. In the present case, dalteparin was changed to argatroban immediately after diagnosis. Interestingly, the patient's subjective and objective symptoms disappeared from that day on. However, the thrombocytopenia persisted and worsened. The main reason for this may have been that the patient could not receive inpatient care because of the coincidence of the SARS-CoV-2 outbreak, so continuous argatroban administration was not possible, resulting in an insufficient dose. It was reported that HIT antibodies disappear spontaneously in 50 to 85 days [12]. However, we considered the use of argatroban itself to be high risk because the patient had a history of right pontine hemorrhage four months before the onset of HIT and a history of percutaneous intraluminal embolization for AAA. Therefore, to minimize the risk of hemorrhagic lesions associated with argatroban, simple PE therapy was performed to achieve the early reduction of HIT antibodies [13]. Although PE therapy for HIT is not a well-established therapy with sufficient evidence, there are many reports of its effectiveness [14,15]. In the present case, after two courses of PE therapy, the thrombocytopenia improved and the patient was in remission. There were no side effects or bleeding lesions throughout the treatment.

## Conclusions

Dialysis patients who receive heparin routinely are at high risk for HIT, but its incidence is high during the induction phase and drastically decreases during the maintenance phase. We experienced a rare case of HIT after four years of the hemodialysis induction, diagnosed using HIT antibodies assay and clinical scores, which led to remission with immediate discontinuation of heparin, administration of argatroban, and simple PE. Because of its severity and mortality, HIT should be considered in the differential diagnosis of thrombocytopenia even during maintenance dialysis.
